# A new treatment for breast cancer using a combination of two drugs: AZD9496 and palbociclib

**DOI:** 10.1038/s41598-023-48305-z

**Published:** 2024-01-15

**Authors:** Ophir Nave, Yehuda Shor, Raziel Bar, Eliezer Elimelech Segal, Moriah Sigron

**Affiliations:** 1 Department of Mathematics, Faculty of Science, Jerusalem College of Technology (JCT), Academic Level Centre, Jerusalem, Israel; 2 Department of Computer Science, Jerusalem College of Technology (Mivchar), Jerusalem, Israel

**Keywords:** Cancer, Computational biology and bioinformatics

## Abstract

In this study, we examined a mathematical model of breast cancer (BC) treatment that combines an oral oestrogen receptor inhibitor, AZD9496 with Palbociclib, a selective inhibitor of cyclin- dependent kinases CDK4 and CDK6. Treatment is described by analytical functions that enable us to control the dosage and time interval of the treatment, thus personalising the treatment for each patient. Initially, we investigated the effect of each treatment separately, and finally, we investigated the combination of both treatments. By applying numerical simulations, we confirmed that the combination of AZD9496 with palbociclib was the optimal treatment for BC. The dosage of AZD9496 increased and decreased throughout the treatment period, while the intervals were constant between treatments. Palbociclib changed almost cyclically, whereas the time intervals remained constant. To investigate the mathematical model, we applied the singularly perturbed homotopy analysis method, which is a numerical algorithm. The significant advantage of this method is that the mathematical model does not have to contain a small parameter (as is standard in perturbation theory). However, it is possible to artificially introduce a small parameter into the system of equations, making it possible to study the model using asymptotic methods.

## Introduction

Breast cancer (BC) is the most common malignancy in women. BC is a malignant tumour that develops from the lining cells (“epithelial cells”) of the system that produces and/or transports milk to the nipple: if the tumour develops from cells of the clusters that produce milk, it is called a “lobular tumour”, and if it originates from the lining cells of the ducts that carry milk to the nipple, it is called a “tubular tumour”. The latter is the most common form (85$\%$) of breast tumour. This tumour is considered the most common malignant disease in women worldwide, but it may also develop in men at a frequency of 1:100 compared with that in women^1–4^.

Increasing awareness of the benefits of early detection of the disease, together with an improvement in the understanding of the disease and treatments, allows for a high rate of cure for those diagnosed^5,6^. It should be remembered that the term “BC” includes a collection of several groups of tumours originating from the breast that differ in risk factors, oestrogen receptor expression, Her2 protein expression, multiple gene expression, and sensitivity to anti-cancer treatments^7^. Therefore, when a malignant breast tumour is diagnosed, the group it belongs to is determined. This association affects the assessment of the disease recurrence risk and therapeutic recommendations^8^.

In general, cancer occurs when multiple changes accumulate in the genetic material of the cell, resulting in the uncontrolled division of diseased cells, metabolic disorders, evasion of the body’s immune system, and other defence mechanisms, which are designed to identify and destroy damaged cells^9^. The damaged cells often divide faster than healthy cells in the environment, and they can form a mass, grow at the expense of healthy tissue, spread through the lymphatics to regional lymph nodes (for example, in breast tumours, the malignant cells may spread to the glands in the armpit), and even sow cells in the bloodstream that will deposit into other organs of the body, such as the bones or the liver, growing and forming tumorous lumps called “metastases”^10^.

Metastatic BC is the term used when a tumour spreads to distant locations. Recent studies have shown that verifying the suitability of drug treatments for metastatic BC can reduce the need for chemotherapy by approximately 50$\%$^11–16^. Currently, there are many options for the treatment of metastatic BC, including chemotherapy, target-oriented biological drugs, and the promising new generation of immunotherapy.

In this study, we focus on a treatment that combines AZD9496, an oral oestrogen receptor inhibitor that blocks the growth of ER- and ESR1-positive breast tumours in preclinical models, with palbociclib, a selective inhibitor of the cyclin-dependent kinases CDK4 and CDK6^17–21^.

The growing awareness of the disease, responsiveness to early detection, and improvements in planning and carrying out treatments according to the characteristics of the tumour have led to a significant increase in the cure rate.

To investigate the nonlinear ODE system, we combined two semi-analytical methods: the homotopy analysis method (HAM) and the method of integral invariant manifold (MIM). HAM employs the concept of homotopy from topology to generate a convergent series solution for nonlinear systems. This is a mathematical technique for solving a system of differential equations without a small parameter appearing in the system^22^.

MIM is a technique used to simplify the study of reaction mechanisms using dynamic systems, as proposed in^23,24^.

### Mathematical models of BC

In recent years, global scientists from various fields, such as physics and mathematics, have integrated their research into applications in medicine and cancer research in particular^25–28^. The main aim is to devise a mathematical model (usually a system of nonlinear ODEs or PDEs) that considers various parameters related to the human body, such as different cells from the immune system and the interaction of these parameters with the tumour itself and with drug treatment. By solving a mathematical model numerically or analytically, it is possible to obtain the size of the tumour as a function of time, which is one of the most significant pieces of data in cancer research. As can be seen from many diverse studies, mathematical models can successfully and accurately predict the size of tumours^29–32^.

There are several significant advantages of using mathematical models in cancer research. The main advantage is that if one wants to adjust the treatment personally (such as the dosage of the drug and the time of administration of the treatment), there is no need to perform experiments on animals or humans, but simply change the parameters of the mathematical model. Another advantage is that an increasing number of equations related to the aforementioned interactions can be added, making the mathematical model more accurate. For example, drug treatment can be described by an explicit function that controls the doses of the drugs and changes the treatment administration times. Another significant advantage is the combination of machine learning (ML) and mathematical models; therefore, a very large dataset can be considered.

Here, we present important examples of BC research using mathematical models.

BC is the most common malignancy in western countries. When the disease is diagnosed in its early stages, the chance of a cure increases to approximately 90$\%$. It appears that the number of women who recover from BC is constantly increasing owing to early detection, improvements in treatment methods, and the widespread increase in global awareness. An example of this success is a 2018 study that included 699 women aged 18-85 with AH; the researchers^33^ created a three-variable model aimed at predicting the risk of developing BC among women with atypical hyperplasia (AH) at biopsy. The three variables were age at the time of biopsy, age at the time of biopsy squared, and the number of AH foci. Women with AH identified on breast biopsy have an increased cumulative risk of developing BC, but the risk prediction models that exist today do not provide an accurate personalised assessment of the risk in this group. In this study, we analysed breast disease (benign growth) to develop and validate a BC risk prediction model that is specifically tailored to women with AH, called AH-BC. Researchers have created a new model that could more accurately predict the risk of BC in women with AH. After 10 years, the AH-BC model demonstrated good discrimination and calibration and was also valid when tested on an external model.

The next example is a study presented by^10^ which describes the combination of a mathematical model describing chemotherapy treatment for BC with a ML algorithm to increase the performance in predicting tumour size using a five-step procedure. The focus of this research involves extracting the numerical solutions obtained from the mathematical model and integrating them into ML as additional data. The authors of this study established this procedure in four cohorts of women of different ages with BC who received chemotherapy. A performance comparison among the algorithms showed that the root mean square error of the linear regression decreased with the addition of the mathematical results, and the accuracy of prediction and the F1-scores increased with the addition of the mathematical model to the neural network.

Another study on mathematical models for BC research was presented by^34^ in 2021. The authors developed a mathematical model to investigate the interaction between immune cells and BC tumour development. The main conclusion of this study, based on the application of numerical simulations to a mathematical model, demonstrated the direct effects of macrophages and adipocytes on cancer cell growth. In addition, interactions among immune cells can affect the immunological responses in various cancer types.

## Formulation of the system of equations

In this section, we present a mathematical model^35^ for ER-positive BC treatment using two different treatments: AZD9496 and palbociclib. We propose a new personalised treatment based on analytical functions which depend on two parameters: the dosage of medicine and the time interval between treatments. These two parameters enable us to control the treatments such that we can change the dosage and time interval depending on the tumour size. For this purpose, we developed an ODE equation for the function of treatments as a function of tumour size. The solution profiles of these equations show the dosage and time interval as a function of the tumour size at each given time.

The dynamical variables of the model are as follows: $C_{C}$ [*cell*] is the MCF-7 tumour cell population, $N_{K}$ $[cellL^{-1}]$, is the natural killer (NK) cell population, $W_{BC}$ $[cellL^{-1}]$ is the white blood cell (WBC) population, $C_{TL}$ $[cellL^{-1}]$ is the cytotoxic T cell (CTL) population, $A^{nc}_{ZD}$ [*mg*] is the AZD9496 not in circulation, $A^{c}_{ZD}$ [*mg*] is the AZD9496 in circulation, $P^{nc}_{a}$ [*mg*] is the palbociclib not in circulation, $P^{c}_{a}$ [*mg*] is the palbociclib in circulation, and $\mathcal {F}$ [*mg*] and $\mathcal {H}$ [*mg*] are functions of the treatment of AZD9496 and palbociclib, respectively ($q_{A_{ZD}}$ is the amount of AZD9496 and $q_{P_{a}}$ is the amount of palbociclib). The mathematical model is a system of ordinary differential equations, nonlinear first order in the form of

1 $$\begin{aligned} \frac{dC_{C}}{dt}= C_{c}\left( ae^{-\alpha _{9}P^{c}_{a}}+\frac{ce^{-\alpha _{10}A^{c}_{ZD}}EC_{c}}{1+\alpha _{1}E+\beta _{1}C^{2}_{C}}\right) \left( 1-\frac{C_{c}}{K}\right) -\frac{p_{1}C_{C}N^{2}_{K}}{1+\alpha _{2}C_{C}+\beta _{2}N^{2}_{K}}-\frac{p_{6}C^{2}_{C}C_{TL}}{1+\alpha _{6}C^{2}_{C}+\beta _{6}C_{TL}}, \end{aligned}$$

2 $$\begin{aligned} \frac{dN_{K}}{dt} = eW_{BC}-fN_{K}-p_{2}N_{K}C_{C}+\frac{p_{3}N_{K}C_{C}}{1+\alpha _{3}C_{C}+\beta _{3}N_{K}}, \end{aligned}$$

3 $$\begin{aligned} \frac{dW_{BC}}{dt} = \alpha -\beta W_{BC}, \end{aligned}$$

4 $$\begin{aligned} \frac{dC_{TL}}{dt}= & {} \left( \frac{C_{C}K_{L}-C_{TL}C_{C}}{K_{L}(\alpha _{5}+C_{C})}\right) \left( p_{4}L_{N}+\frac{p_{5}I}{\alpha _{4}+I}C_{TL}\right) -dC_{TL}, \end{aligned}$$

5 $$\begin{aligned} \frac{dA^{nc}_{ZD}}{dt}= & {} -\alpha _{7}A^{nc}_{ZD}+\mathcal {F}(t, q_{A_{ZD}}), \end{aligned}$$

6 $$\begin{aligned} \frac{dA^{c}_{ZD}}{dt} = \alpha _{7}A^{nc}_{ZD}-\beta _{7}A^{c}_{ZD}, \end{aligned}$$

7 $$\begin{aligned} \frac{dP^{nc}_{a}}{dt}= & {} -\alpha _{8}P^{nc}_{a}+\mathcal {H}(t, q_{P_{a}}), \end{aligned}$$

8 $$\begin{aligned} \frac{dP^{c}_{a}}{dt} = \alpha _{8}P^{nc}_{a}-\beta _{8}P^{c}_{a}, \end{aligned}$$

9 $$\begin{aligned} \frac{d\mathcal {F}}{dt}= & {} \mathcal {F}(t, q_{A_{ZD}})^{\epsilon _{1}}\cdot C_{C}-\mathcal {F}(t, q_{A_{ZD}})^{\epsilon _{2}}, \end{aligned}$$

10 $$\begin{aligned} \frac{d\mathcal {H}}{dt}= & {} \mathcal {H}(t, q_{P_{a}})^{\epsilon _{3}}\cdot C_{C}-\mathcal {H}(t, q_{P_{a}})^{\epsilon _{4}}. \end{aligned}$$

11 $$\begin{aligned} E(t) = \tilde{E}(t-n\tau ), t\in \left[ n\tau , (n+1)\tau \right] , n=1, 2, 3... \end{aligned}$$

The initial conditions of the model at $t=0$ are

12 $$\begin{aligned}{} & {} C_{C}=8.72\cdot 10^{7},\quad N_{K}=2.5\cdot 10^{8},\quad W_{BC}=4.3\cdot 10^{9},\quad C_{TL}=6.6\cdot 10^{8},\quad A^{nc}_{ZD}=0,\nonumber \\{} & {} \quad A^{c}_{ZD}=0,\quad P^{nc}_{a}=0,\quad P^{c}_{a}=0,\quad \mathcal {F}(0, q_{A_{ZD}})=q^{1}_{0},\quad \mathcal {H}(0, q_{P_{a}})=q^{2}_{0}. \end{aligned}$$

In the next section, we apply the singularly perturbed homotopy analysis method (SPHAM) to investigate the mathematical model above.

## Combination of HAM and MIM

In this section, we present HAM, MIM, and a combination of these two semi-analytical methods.

### HAM

In this section we introduce the HAM procedure as present in^36^.

Given a nonlinear system of differential equations in the form of

13 $$\begin{aligned} N(u(\vec {r}, t))=0. \end{aligned}$$

where *N* is a nonlinear operator, $\vec {r}$ is a vector of spatial variables, *t* is time, and *u* is an unknown function. By generalising the traditional concept of homotopy^37^, constructed the so-called zero-order deformation equation

14 $$\begin{aligned} (1-p)\ell \left[ \Psi (\vec {r}, t; p)-u_{0}(\vec {r}, t)\right] =\hslash H(\vec {r}, t) N(\Psi (\vec {r}, t; p)), \end{aligned}$$

where $\hslash$ is a nonzero auxiliary parameter, *H* is an auxiliary function, $\ell$ is an auxiliary linear operator, $u_{0}(\cdot )$ is the initial guess for $u(\cdot )$, and $\Psi$ is an unknown function. The degrees of freedom are used to choose the initial guess, auxiliary linear operator, auxiliary parameter, and auxiliary function, *H*. To obtain the corresponding *m*th-order deformation of the HAM, we define the vector as follows:

15 $$\begin{aligned} \vec {u_{n}}(\vec {r}, t)=\{u_{0}(\vec {r}, t), u_{1}(\vec {r}, t),\ldots ,u_{n}(\vec {r}, t)\}. \end{aligned}$$

Differentiating Eq. (14) *m* times with respect to the embedding parameter *p*, setting $p=0$, and finally dividing the terms by *m*!, we obtain the *m*th-order deformation equation in the form

16 $$\begin{aligned} \ell \left[ u_{m}(\vec {r}, t)-\mathcal {H}_{m}u_{m-1}(\vec {r}, t)\right] =\hslash H(\vec {r}, t)R_{m}(u_{m-1}(\vec {r}, t)), \end{aligned}$$

where,

17 $$\begin{aligned} R_{m}(u_{m-1}(\vec {r}, t))=\frac{1}{(m-1)!}\frac{\partial ^{m-1}N(\Psi (\vec {r}, t; p))}{\partial p^{m-1}}|_{p=0}. \end{aligned}$$

Applying the inverse operator $\ell ^{-1}(\cdot )$ to both sides of Eq. (16), we obtain

18 $$\begin{aligned} u_{m}(\vec {r}, t)=\mathcal {H}_{m}u_{m-1}(\vec {r}, t)+\hslash \ell ^{-1}\left[ H(\vec {r}, t)R_{m}(u_{m-1}(\vec {r}, t)) \right] . \end{aligned}$$

Thus, it is easy to obtain $u_{m}$ for $m\ge 1$ at *m*th-order and finally obtain the solution of (13) as follows:

19 $$\begin{aligned} u(\vec {r}, t)=\sum _{n=0}^{m}u_{n}(\vec {r}, t). \end{aligned}$$

In our model, we choose the initial estimates to be $\theta _{g}(0)=0$ and $r_{m}(0)=1$ which satisfy the initial conditions. The linear operator is as follows:

20 $$\begin{aligned} \ell =\frac{d}{d\tau }(\cdot ), \end{aligned}$$

with the property $\ell (c_{1}\tau +c_{2})=0$, where $c_{1}$ and $c_{2}$ are the constants of integration.

Following the above procedure, we define the series’ for the dynamic variables $C_{C}$, $N_{K}$, $W_{BC}$, $C_{TL}$, $A^{nc}_{ZD}$, $A^{c}_{ZD}$, $P^{nc}_{a}$, and $P^{c}_{a}$ as

21 $$\begin{aligned} \Psi _{1}= & C_{C}^{0}(\tau )+\sum _{j=1}^{\infty }C_{C}^{j}(\tau , \hslash )p^{j},\quad \Psi _{2}=N_{K}^{0}(\tau )+\sum _{j=1}^{\infty }N_{K}^{j}(\tau , \hslash )p^{j},\nonumber \\ \Psi _{3}= & W_{BC}^{0}(\tau )+\sum _{j=1}^{\infty }W_{BC}^{j}(\tau , \hslash )p^{j},\quad \Psi _{4}=C_{TL}^{0}(\tau )+\sum _{j=1}^{\infty }C_{TL}^{j}(\tau , \hslash )p^{j},\nonumber \\ \Psi _{5}= & {A^{nc}_{ZD}}^{0}(\tau )+\sum _{j=1}^{\infty }{A^{nc}_{ZD}}^{j}(\tau , \hslash )p^{j},\quad \Psi _{6}={A^{c}_{ZD}}^{0}(\tau ) +\sum _{j=1}^{\infty }{A^{c}_{ZD}}^{j}(\tau , \hslash )p^{j},\nonumber \\ \Psi _{7}= & {} P^{nc}_{a}(\tau )+\sum _{j=1}^{\infty }{P^{nc}_{a}}^{j}(\tau , \hslash )p^{j},\quad \Psi _{8}={P^{c}_{a}}^{0}(\tau )+\sum _{j=1}^{\infty }{P^{c}_{a}}^{j}(\tau , \hslash )p^{j}. \end{aligned}$$

Based on the above notation, we let

22 $$\begin{aligned}{} & {} \ell \left[ C_{C}^{m}(\tau , \hslash )-\mathcal {H}_{m}C_{C}^{m-1}(\tau , \hslash )\right] =\hslash R_{m_{C_{C}}},\nonumber \\{} & {} \quad \ell \left[ N_{K}^{m}(\tau , \hslash )-\mathcal {H}_{m}N_{K}^{m-1}(\tau , \hslash )\right] =\hslash R_{m_{N_{K}}},\nonumber \\{} & {} \quad \ell \left[ W_{BC}^{m}(\tau , \hslash )-\mathcal {H}_{m}W_{BC}^{m-1}(\tau , \hslash )\right] =\hslash R_{m_{W_{BC}}},\nonumber \\{} & {} \quad \ell \left[ C_{TL}^{m}(\tau , \hslash )-\mathcal {H}_{m}C_{TL}^{m-1}(\tau , \hslash )\right] =\hslash R_{m_{C_{TL}}},\nonumber \\{} & {} \quad \ell \left[ {A^{nc}_{ZD}}^{m}(\tau , \hslash )-\mathcal {H}_{m}{A^{nc}_{ZD}}^{m-1}(\tau , \hslash )\right] =\hslash R_{m_{A^{nc}_{ZD}}},\nonumber \\{} & {} \quad \ell \left[ {A^{c}_{ZD}}^{m}(\tau , \hslash )-\mathcal {H}_{m}{A^{c}_{ZD}}^{m-1}(\tau , \hslash )\right] = \hslash R_{m_{A^{c}_{ZD}}},\nonumber \\{} & {} \quad \ell \left[ {P^{nc}_{a}}^{m}(\tau , \hslash )-\mathcal {H}_{m}{P^{nc}_{a}}^{m-1}(\tau , \hslash )\right] = \hslash R_{m_{P^{nc}_{a}}},\nonumber \\{} & {} \quad \ell \left[ {P^{c}_{a}}^{m}(\tau , \hslash )-\mathcal {H}_{m}{P^{c}_{a}}^{m-1}(\tau , \hslash )\right] =\hslash R_{m_{P^{c}_{a}}}. \end{aligned}$$

The set of equations (21) defines the analytical solutions as a power series of the dynamic variables of the system (1)–(8) and the set of equations (21) defines the linear operator.

#### MIM

In this section, we introduce the primary theory underlying MIM. To apply the MIM, the system of differential equations must be in the form of a singular perturbed system:

23 $$\begin{aligned} \epsilon \cdot \frac{d\vec {x}}{dt}= & {} \vec {\mathcal {T}}_{fast}\left( \vec {x}, \vec {y}, \epsilon \right) \nonumber \\ \frac{d\vec {y}}{dt}= & {} \vec {\mathcal {Q}}_{slow}\left( \vec {x}, \vec {y}, \epsilon \right) , \end{aligned}$$

where $0<\epsilon<<1$ is the small parameter of the system, $\vec {x}=(x_{1},\ldots ,x_{k_{fast}})\in \mathbb {R}^{k_{fast}}$, and $\vec {y}=(y_{1},\ldots ,y_{l_{slow}})\in \mathbb {R}^{l_{slow}}$ are the fast and slow dynamic variables, respectively, of the system ($k_{fast}+l_{slow}=n$). The functions $\vec {\mathcal {T}}$ and $\vec {\mathcal {Q}}$ are assumed to be $C^{\infty }$ of $\vec {x}$, $\vec {y}$ and $\epsilon$ in $(V\times U)\times I$ where *V* and *U* are open subsets of $\mathbb {R}^{k_{fast}}$ and $\mathbb {R}^{l_{slow}}$, respectively. *I* is an open interval containing the small parameter $\epsilon$.

In general, when dealing with models describing natural phenomena, the small parameter does not appear explicitly in the system of equations, but in a hidden form; that is, the hierarchy of the mathematical model is hidden.

To apply MIM, the equations for *m* deformations of the linear operator can be rewritten as follows:

24 $$\begin{aligned} F_{0}\left( x_{fast}^{1},\ldots ,x_{k_{fast}}^{m-1},y_{slow}^{1},\ldots ,y_{slow}^{m-1}\right) + \gamma F_1 \left( x_{fast}^{m-1} \right) +\gamma ^2 F_2 \left( x_{fast}^{m-1}\right) =0 , \end{aligned}$$

where $F_{0}, F_{1},$ and $F_{2}$ are the corresponding terms in Eq. (23).

We obtain the second order by the small parameter $\gamma$ perturbation of the equation

25 $$\begin{aligned} F_0 \left( x_{fast}^{1},\ldots ,x_{fast}^{m-1},y_{slow}^{1},\ldots ,y_{slow}^{m-1}\right) =0. \end{aligned}$$

According to the standard regular perturbation theory, the previous equation is a zero-order approximation of the perturbed equation^37^ (i.e., terms with $\gamma$ and $\gamma ^{2}$ vanish). The first-order approximation is as follows:

26 $$\begin{aligned} F_0 \left( x_{fast}^{1},\ldots ,x_{fast}^{m-1},y_{slow}^{1},\ldots ,y_{slow}^{m-1}\right) +\gamma F_1 \left( x_{fast}^{m-1}\right) =0. \end{aligned}$$

This procedure enabled us to expand the fast variable to a power series of small system parameters.

#### Combination of HAM with MIM

In this section, we present a combination of a HAM and MIM called SPHAM.

A combination of these two methods is necessary because the mathematical model presented in the system of Eqs. (1)–(11) does not have an explicit hierarchy; that is, the hierarchy of the system is hidden; therefore, we cannot apply MIM separately. However, HAM does not require a small parameter of the system; the small parameter artificially enters the system of differential equations, which is problematic because not all variables of the mathematical model are fast. Therefore, we developed an algorithm that combined these methods.

**The algorithm for the combination of HAM with MIM:**

1. Substitute $\gamma =0=\gamma ^{2}$ into Eq. (24),
2. Substitute $m=1$ in Eq. (17) (the expression of $R_{m_{u}}$, where *u* is the dynamic variable of the system). From the expression $R_{m_{u_{1}}}$ (where 1 is the first variable of the system), we obtain the expression $u_{2}^{0}$ (where 2 is the second variable of the system)
3. Substitute the initial condition $u_{2}^{0}=u_{2}(0)$ in step 2 and obtain an equation for $u_{1}^{0}$:
4. Analytically solve the equation for $u_{1}^{0}$.
5. Substitute $u_{1}^{0}$ and the initial condition $u_{2}^{0}=u_{2}(0)$ into the expressions $R_{1_{u_{1}}}$ and $R_{1_{u_{2}}}$,
6. Repeat steps $1-4$ for the remaining variables and initial conditions of system (1)–(11),
7. Substitute the expressions from Step 6 into the following 1-order deformation:
  27 $$\begin{aligned}{} & {} C_{C}^{1}(\tau , \hslash )=\mathcal {H}_{1}C_{C}^{0}(\tau , \hslash )+\hslash \ell ^{-1}R_{1_{C_{C}}}\left( C_{C}^{0}\right) ,\nonumber \\{} & {} \quad N_{K}^{1}(\tau , \hslash )=\mathcal {H}_{1}N_{K}^{0}(\tau , \hslash )+\hslash \ell ^{-1}R_{1_{N_{K}}}\left( N_{K}^{0}\right) ,\nonumber \\{} & {} \quad W_{BC}^{1}(\tau , \hslash )=\mathcal {H}_{1}W_{BC}^{0}(\tau , \hslash )+\hslash \ell ^{-1}R_{1_{W_{BC}}}\left( W_{BC}^{0}\right) ,\nonumber \\{} & {} \quad C_{TL}^{1}(\tau , \hslash )=\mathcal {H}_{1}C_{TL}^{0}(\tau , \hslash )+\hslash \ell ^{-1}R_{1_{C_{TL}}}\left( C_{TL}^{0}\right) ,\nonumber \\{} & {} \quad {A^{nc}_{ZD}}^{1}(\tau , \hslash )=\mathcal {H}_{1}{A^{nc}_{ZD}}^{0}(\tau , \hslash )+\hslash \ell ^{-1}R_{1_{A^{nc}_{ZD}}}\left( {A^{nc}_{ZD}}^{0}\right) ,\nonumber \\{} & {} \quad {A^{c}_{ZD}}^{1}(\tau , \hslash )=\mathcal {H}_{1}{A^{c}_{ZD}}^{0}(\tau , \hslash )+\hslash \ell ^{-1}R_{1_{A^{c}_{ZD}}}\left( {A^{c}_{ZD}}^{0}\right) ,\nonumber \\{} & {} \quad {P^{nc}_{a}}^{1}(\tau , \hslash )=\mathcal {H}_{1}{P^{nc}_{a}}^{0}(\tau , \hslash )+\hslash \ell ^{-1}R_{1_{P^{nc}_{a}}}\left( {P^{nc}_{a}}^{0}\right) ,\nonumber \\{} & {} \quad {P^{c}_{a}}^{1}(\tau , \hslash )=\mathcal {H}_{1}{P^{c}_{a}}^{0}(\tau , \hslash )+\hslash \ell ^{-1}R_{1_{P^{c}_{a}}}\left( {P^{c}_{a}}^{0}\right) , \end{aligned}$$
8. Repeat steps 1–7 *m* times to obtain the *m*-order deformation of each variable of the mathematical model.
9. Obtain the following explicit expressions:
  28 $$\begin{aligned} u^{m}(\tau , \hslash )=\mathcal {H}_{m}u^{m-1}(\tau , \hslash )+\hslash \ell ^{-1}\left[ R_{m_{u}}(u^{m-1}(\tau , \hslash ))\right] , \end{aligned}$$
  where *u* represents the variables in the system.
10. Sum the expressions from step 9 and obtain the following semi-analytical approximation for the dynamic variables of the mathematical model:
  29 $$\begin{aligned} u=u^{0}+\sum _{m=1}^{M}u^{m}(\tau , \hslash ). \end{aligned}$$.

## Results and discussion

In this section, we investigate the mathematical model (1–11) with the initial conditions (12) which presents BC treatment combining an oral oestrogen receptor inhibitor called AZD9496 with palbociclib, which is a selective inhibitor of the cycling-dependent kinases CDK4 and CDK6 for non-standard protocols, using the functions $\mathcal {F}$ and $\mathcal {H}$ for different doses and time intervals.

palbociclib was administered orally and was absorbed slowly from the intestine in 6–12 h.

We applied a combination of HAM and MIM and obtained the semi-analytical solution profiles of the dynamic variables of the mathematical model (1)-(11) using the parameters presented in Tables 1, 2.

**Figure 1:**
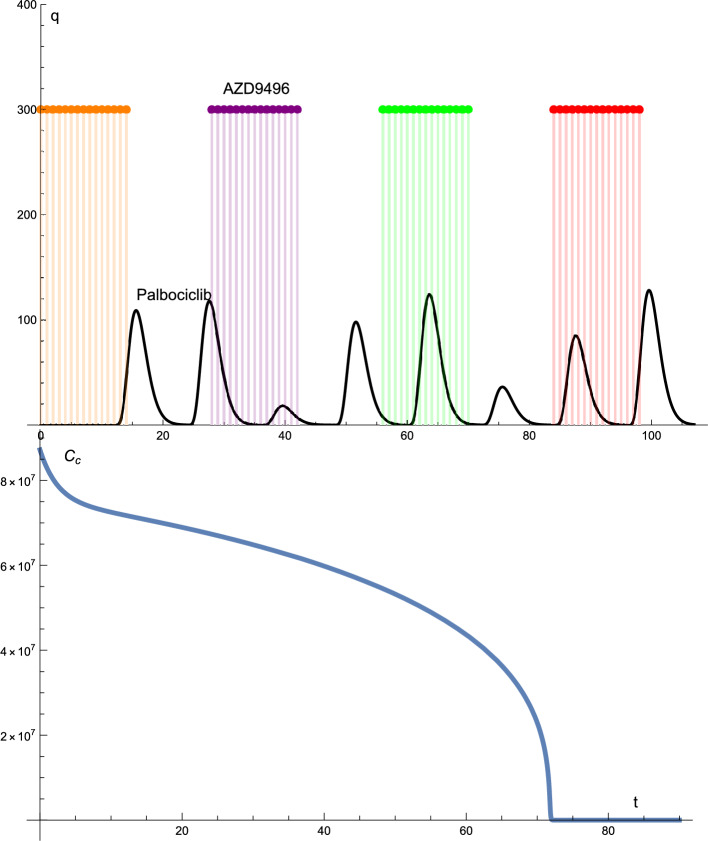
The solutions profiles of the cancer cells and the drugs protocols. The Palbociclib drug given is given arbitrarily i.e., the dose of the drug changes arbitrarily but the time intervals are constant. Whereas the AZD9496 drug is given according to the standard protocol, the dosage and time intervals are constant.

Figure  1 shows the solution profiles of the cancer cells AZD9496 and palbociclib. AZD9496 is administered at a constant dose of 300 mg. One tablet daily for 14 days, followed by 14 days off, for four cycles of administration. Palbociclib is administered arbitrarily, i.e., the dose of the drug changes arbitrarily, and the time interval of the drug is constant. The maximum and minimum doses were 125 mg and 20 mg, respectively. In this case, as can be seen from the solution profile of the cancer cells, the cancer decreased from the initial condition to zero after 72 days.

**Figure 2:**
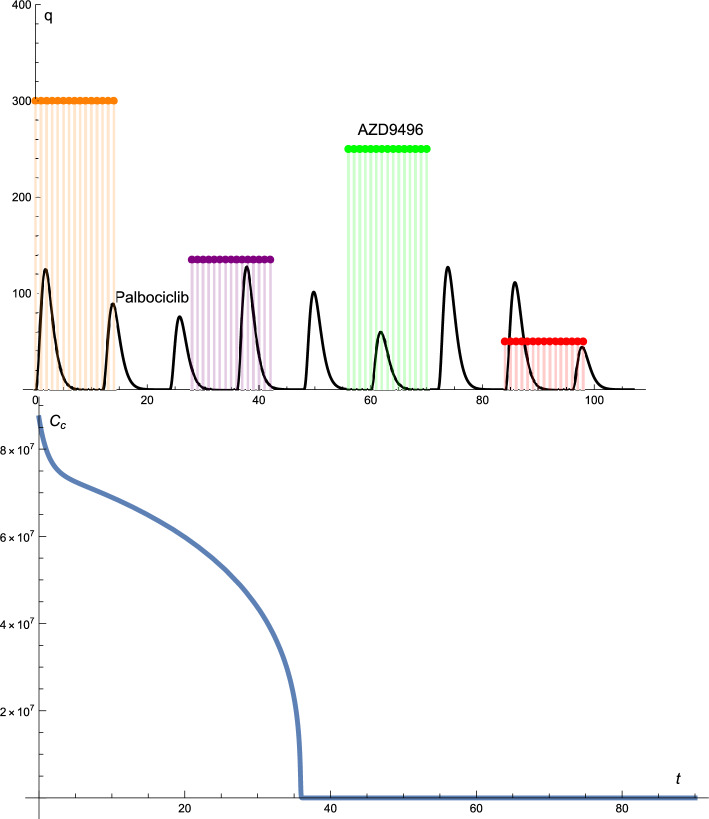
The solutions profiles of the cancer cells and the drug protocols. The AZD9496 drug given is not according to the standard protocol, that is, the dose of the drug changes up and down, whereas the time intervals are constant. The dosage of the Palbociclib drug changes almost cyclic whereas the time intervals are constant.

Figure  2 presents the solution profiles of the cancer cells and drug protocols. The AZD9496 administered did not follow the standard protocol; that is, the dose of the drug was changed, whereas the time intervals were constant. The administration of AZD9496 is as follows: 300 mg every day for 14 days, break for 14 days, 135 mg every day for 14 days, break for 14 days, 250 mg every day for 14 days, break for 14 days, and 50 mg every day for 14 days.

Palbociclib is not a standard protocol. In this case, the drug dosage changed almost cyclically. The time intervals were kept constant. The dose of palbociclib starts at 125 mg, is reduced to 85 mg and then 75 mg, after which the dose again is increased to 125 mg, reduced to 100 mg and then 60 mg, again increased to 125 mg, and finally reduced to 110 mg. At the end of the treatment, the dose of palbociclib was reduced to 40 mg.

In this combination of treatments, we achieved optimal treatment for the cancer cells which were reduced to zero after 32 d of treatment, as can be seen from the solution profile of the cancer cells presented in Fig.  2.

**Figure 3:**
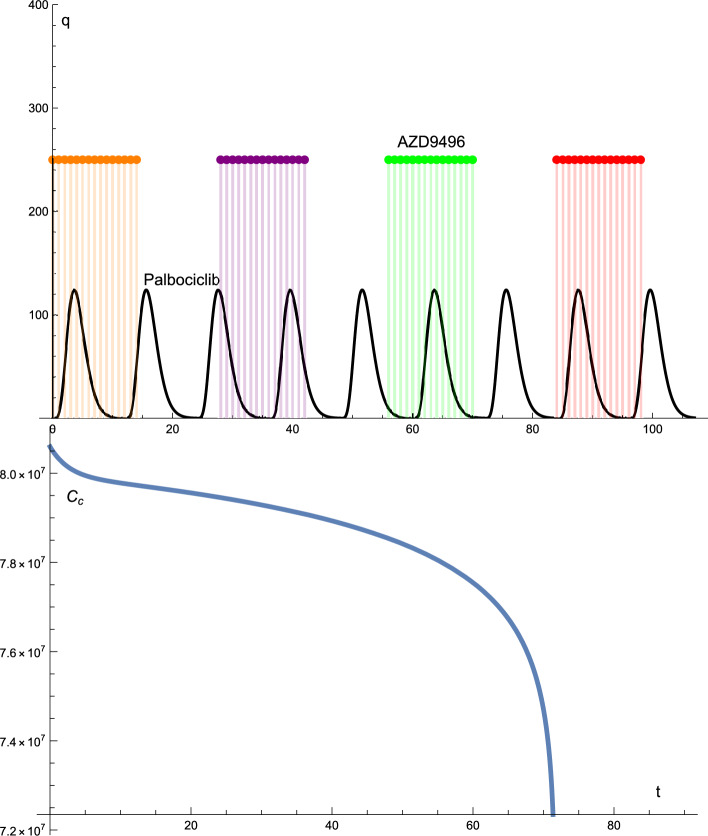
The solutions profiles of the cancer cells and the drugs protocols. The dose of AZD9496 and Palbociclib drugs is constant in terms of the dosage and time intervals.

Figure  3 shows the solution profiles of the cancer cells and drug protocols. The doses of AZD9496 and palbociclib were constant in terms of dosage and time intervals: 250 mg of AZD9496, a cycle of one tablet per day for 14 days and a break of 14 days. The dose of palbociclib was maintained at 125 mg at constant intervals.

In this case, the number of cancer cells decreased from the initial condition to zero after 72 days. The significant difference between Figs.  1 and  3 is that, although both decrease to zero after 72 days, in the last graph, the cancer cells decreases to zero more quickly than those in the previous graph.

**Figure 4:**
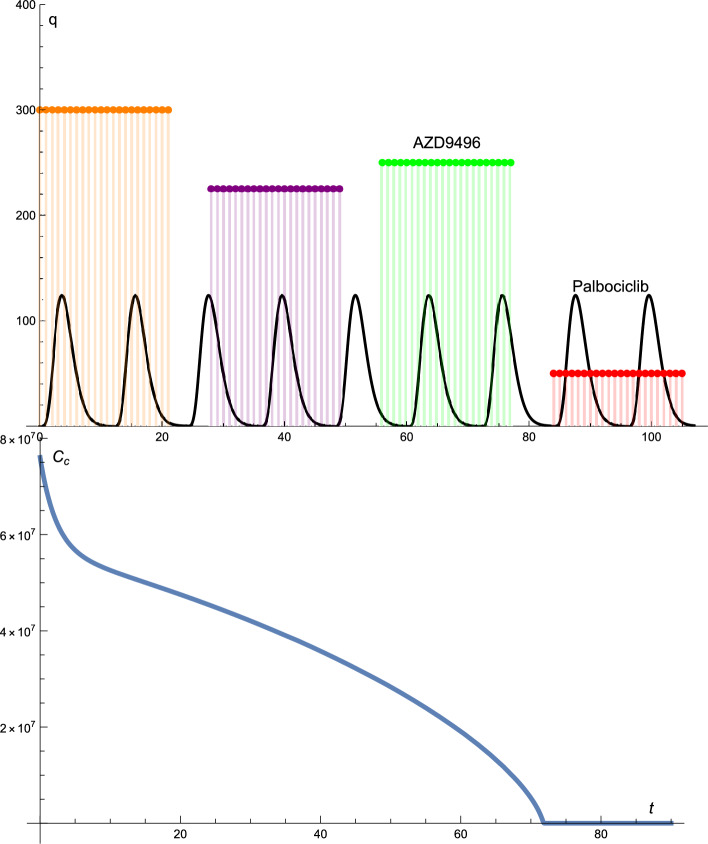
The solutions profiles of the cancer cells and the drugs protocols. The dose and the time intervals of Palbociclib are constant. Whereas the dosage of the AZD9496 drug changes. The dose does not change regularly, that is, it increases and decreases from the maximum 300 mg to the minimum of 50 mg.

Figure  4 presents the solution profiles of the cancer cells and drug protocols. The dose of palbociclib was constant in terms of dosage (125 mg) and time interval.

However, the dose of AZD9496 was altered. The dose did not change regularly; that is, it increased and decreased. At the beginning of the treatment, the doses of AZD9496 were 300, 225, 250, and 50 mg. The drug administration times were different from those in the previous cases. The drug was administered for 21 days, with a break of 7 days and then administered again for 21 days.

In this case, the solution profile of cancer cells decreased rapidly to zero after 72 days. The graph decreases approximately linearly to zero.

**Figure 5:**
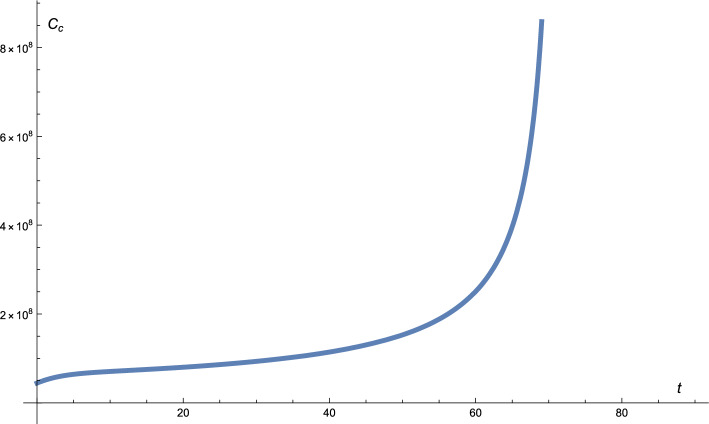
The solution profile of the cancer cells. No treatment.

Figure  5 shows the profiles of untreated cancer cells. It is clear that in this case, cancer cells grow rapidly.

**Figure 6:**
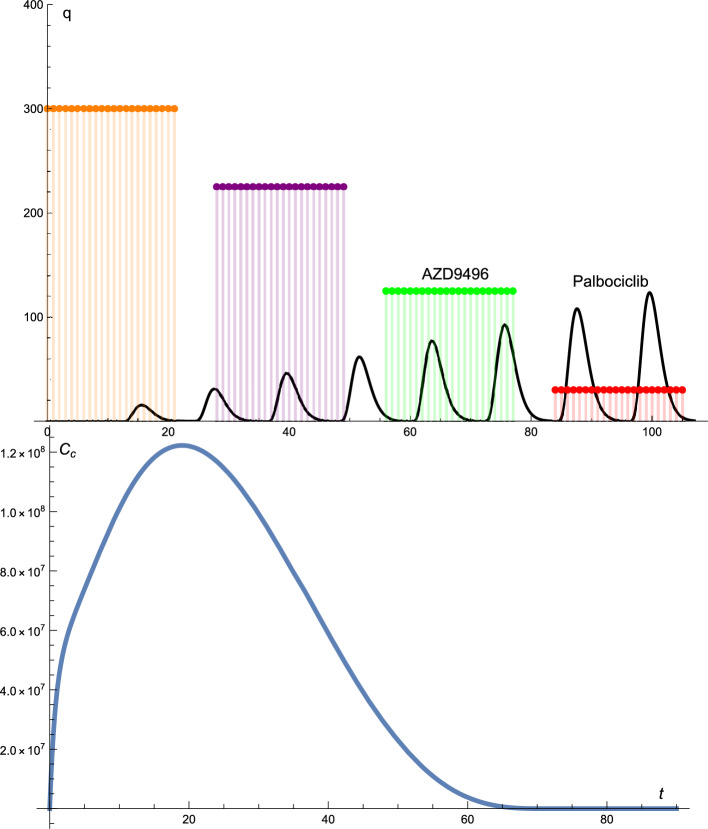
The solutions profiles of the cancer cells and the drugs protocols. The dose of the drug Palbociclib is constantly increased while the dose of the drug AZD9496 is constantly decreased. Both of the drug’s time intervals are constant.

Figure  6 presents the solution profiles of the cancer cells and drug protocols. The dose of palbociclib increased from 20 to 125 mg. However, the dose of AZD9496 constantly decreased, starting at 300 to 225, 125, and finally, 50 mg. In this case, we see that the cancer cells, at the beginning of the treatment, started to increase for 23 days from the initial conditions up to $1.2\cdot 10 ^{8}$ and then started to decrease for 45 days until they reached zero. The biological explanation for this phenomenon is that at the beginning of treatment, the dose of palbociclib was very low (20 mg) and then gradually increased.

**Figure 7:**
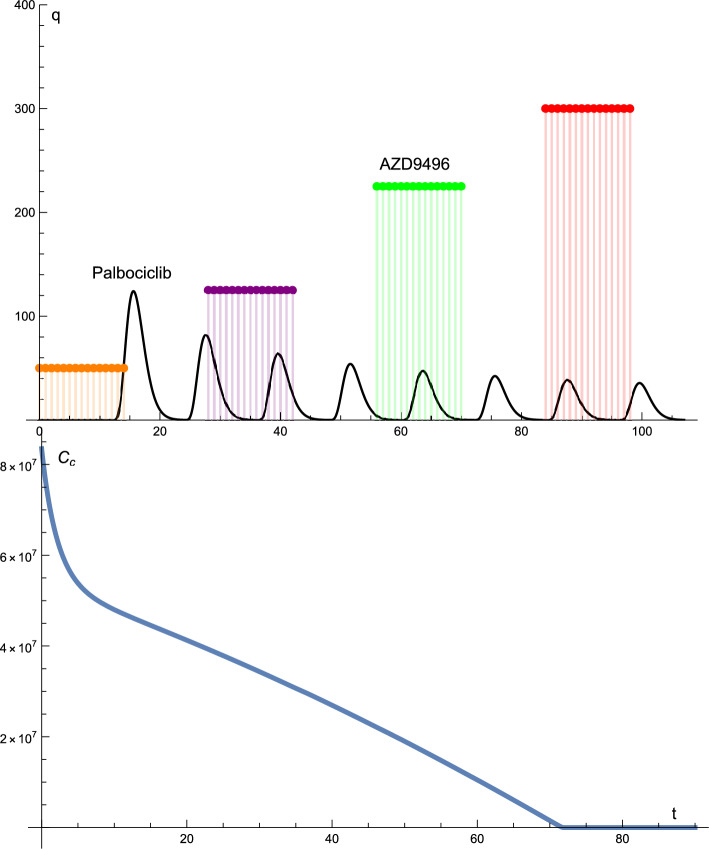
The solutions profiles of the cancer cells and the drugs protocols. The dose of the drug Palbociclib is constantly decreased while the dose of the drug AZD9496 is constantly increased. Both of the drug’s time intervals are constant.

Figure  7 presents the solution profiles of the cancer cells and drug protocols. In contrast to Fig.  6, in this case, the dose of palbociclib constantly decreased, starting from 125 mg, whereas the dose of AZD9496 constantly increased from 50 to 125, 225, and finally 300 mg. This case is also optimal, as can be seen from the graph of the cancer cells. The number of cancer cells decreased to zero in an approximation of a linear function relatively quickly.

## Conclusions

In this study, we present a combination of two semi-analytical methods, HAM and MIM, to investigate the mathematical model of a BC treatment that combines an oral oestrogen receptor inhibitor called AZD9496 with palbociclib, a selective inhibitor of the cyclin-dependent kinases CDK4 and CDK6. A significant advantage of combining these two semi-analytical methods is that the mathematical model does not explicitly contain a small system parameter. By combining these methods, we revealed the hierarchy of the system of equations, which allowed us to divide the mathematical model into fast and slow subsystems. Thus, we studied only the fast system, while the slow system was frozen. In addition, we proposed two explicit analytical functions of the drugs, AZD9496 and palbociclib which are time-and dose-dependent functions of these drugs. This allowed us to control the dose and time intervals of treatment. Thus, we used different dose combinations of both drugs and found that there are two optimal combinations for the treatment of BC. One is that AZD9496 was not administered according to the standard protocol; that is, the dose of the drug changed cyclically. Additionally, the dose of palbociclib varied. The dose did not change regularly; that is, it increased and decreased. Second, the dose of AZD9496 constantly decreases while the dose of palbociclib constantly increases.

## Figures and tables

In this section we present the values of the parameters used for numerical siulation apply to the mathematical model (1–11). In addition we present the solution profiles of the dynamical variables of the system.

**Table 1:** Parameter values using in the mathematical model (1–11).

Parameter	Value	Units	Description
K	$10^{9}$	cell	Tumor cell carrying capacity
c	0.00147	L ${\hbox {Cell}}^{1}$ $Day^{-1}$ $pmol^{-1}$	Tumor growth rate induced by E2
$\alpha _{10}$	0.2263	${\hbox {mg}}^{-1}$	Tumor growth inhibition by AZD9496
$\alpha _{1}$	0.507	L ${\hbox {pmol}}^{-1}$	Half saturation constant
$\beta _{1}$	7.08$\cdot$ $10^{-8}$	${\hbox {Cell}}^{-2}$	Half saturation constant
$p_{1}$	8.7$\cdot$ $10^{-4}$	${\hbox {L}}^{2}$ $Cell^{-2}$ $Day^{-1}$	NK induced tumor death
$\alpha _{2}$	7 $\cdot$ $10^{6}$	${\hbox {Cell}}^{-1}$	Half saturation constant
$\beta _{2}$	5.4$\cdot$ $10^{-5}$	${\hbox {L}}^{2}$ $Cell^{-2}$	Half saturation constant
$\beta$	6.3$\cdot$ $10^{-3}$	${\hbox {Day}}^{-1}$	WBC death rate
$\alpha$	5$\cdot$ $10^{7}$	Cell ${\hbox {L}}^{-1}$ $Day^{-1}$	WBC production rate
e	0.00486	${\hbox {Day}}^{-1}$	Fraction of WBCs becoming NK cells
f	0.0693	${\hbox {Day}}^{-1}$	NK cell death rate
$p_{2}$	3.42 $\cdot$ $10^{-6}$	Cell ${\hbox {Day}}^{-1}$	NK cell inactivation by tumor cells
$p_{3}$	1.87$\cdot$ $10^{-8}$	${\hbox {Cell}}^{-1}$ $Day^{-1}$	NK cell recruitment rate
$\alpha _{3}$	1.6$\cdot$ $10^{-5}$	${\hbox {Cell}}^{-1}$	Half saturation constant
$\beta _{3}$	3.27	L ${\hbox {Cell}}^{-1}$	Half saturation constant

**Table 2:** Parameter values using in the mathematical model (1–11).

Parameter	Value	Units	Description
$p_{6}$	2.04$\cdot 10^{-3}$	L ${\hbox {Cell}}^{-2}$ $Day^{-1}$	CTL induced tumor death
$\alpha _{6}$	0.268	${\hbox {Cell}}^{2}$	Half saturation constant
$\beta _{6}$	4341	L ${\hbox {Cell}}^{-1}$	Half saturation constant
$K_{L}$	8 $\cdot 10^{8}$	${\hbox {Cell}}\,\, {\hbox {L}}^{-1}$	CTL carrying capacity
${\hbox {p}}_{5}$	4.14	L ${\hbox {Cell}}^{-2}$ $Day^{-1}$	CTL growth rate induced by IL-2
d	0.41	${\hbox {Day}}^{-1}$	CTL death rate
$\alpha _{5}$	1000	Cell	Half saturation constant
$\alpha _{7}$	24.3659	${\hbox {Day}}^{-1}$	Absorption rate of AZD9496
$\beta _{7}$	4.7541	${\hbox {Day}}^{-1}$	Elimination rate of AZD9496
${\hbox {p}}_{4}$	9$\cdot 10^{-5}$	${\hbox {Day}}^{-1}$	Fraction of naive CTL activated
$\alpha _{4}$	2.3$\cdot 10^-{11}$	g ${\hbox {L}}^{-1}$	Half saturation constant
$L_{N}$	2.3$\cdot 10^{8}$	Cell ${\hbox {L}}^{-1}$	Naive CTL population
I	2.3$\cdot 10^{-11}$	g ${\hbox {L}}^{-1}$	IL-2 concentration
$\beta _{8}$	0.64	${\hbox {Day}}^{-1}$	Elimination rate of palbociclib
$\alpha _{8}$	14.1512	${\hbox {Day}}^{-1}$	Absorption rate of palbociclib
$\epsilon _{i}$		dimensionless	Free parameter

## Data Availability

All authors declare that all data generated or analysed during this study are included in this published article.
